# *trans*-Diaqua­bis(2,2′-bipyridine-κ^2^ *N*,*N*′)ruthenium(II) bis­(trifluoro­methane­sulfonate)

**DOI:** 10.1107/S1600536808028195

**Published:** 2008-10-11

**Authors:** Hershel Jude, Peter S. White, Dana M. Dattelbaum, Reginaldo C. Rocha

**Affiliations:** a Los Alamos National Laboratory, MPA-CINT, Mail Stop G755, Los Alamos, NM 87545, USA; b University of North Carolina, Department of Chemistry, Chapel Hill, NC 27599-3290, USA; c Los Alamos National Laboratory, DE-9, Mail Stop P952, Los Alamos, NM 87545, USA

## Abstract

The title compound, *trans*-[Ru(bpy)_2_(H_2_O)_2_](CF_3_SO_3_)_2_ (bpy = 2,2′-bipyridine, C_10_H_8_N_2_), crystallized from the decomposition of an aged aqueous solution of a dimeric complex of *cis*-Ru(bpy)_2_ in 0.1 *M* triflic acid. The Ru^II^ ion is located on a crystallographic inversion center and exhibits a distorted octa­hedral coordination with equivalent ligands *trans* to each other. The Ru—O distance is 2.1053 (16) Å and the Ru—N distances are 2.0727 (17) and 2.0739 (17) Å. The bpy ligands are bent, due to inter-ligand steric inter­actions between H atoms of opposite pyridyl units across the Ru center. The crystal structure exhibits an extensive hydrogen-bonding network involving the water ligands and the trifluoromethane­sulfonate counter-ions within two-dimensional layers, although no close hydrogen-bond inter­actions exist between different layers.

## Related literature

For the crystal structures of related compounds, see: Weathers *et al.* (1997 ▶); Durham *et al.* (1980 ▶); Klüfers & Zangl (2007 ▶). For a comparative discussion, see the *Comment* section in the *Supplementary materials*. For the preparation of the title compound, see: Jude *et al.* (2008 ▶); Sullivan *et al.* (1978 ▶). For related literature, see: Walsh & Durham (1982 ▶).
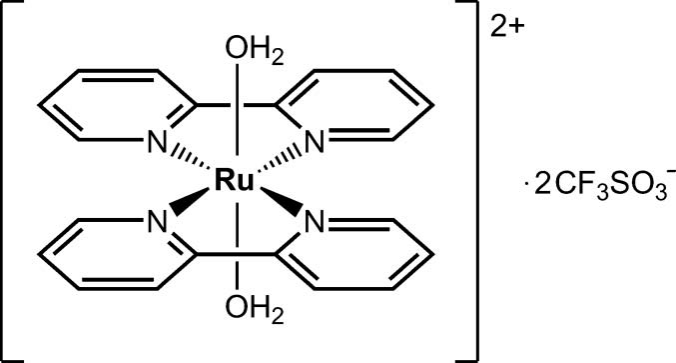


## Experimental

### 

#### Crystal data

[Ru(C_10_H_8_N_2_)_2_(H_2_O)_2_](CF_3_SO_3_)_2_
*M* *_r_* = 747.61
Monoclinic, 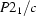

*a* = 8.6569 (5) Å
*b* = 14.1272 (8) Å
*c* = 11.3226 (6) Å
β = 93.095 (3)°
*V* = 1382.71 (13) Å^3^
*Z* = 2
Cu *K*α radiation
μ = 6.88 mm^−1^
*T* = 100 (2) K
0.20 × 0.15 × 0.02 mm

#### Data collection

Bruker SMART APEXII CCD area-detector diffractometer
Absorption correction: numerical followed by *SADABS* (Bruker, 2007 ▶) *T*_min_ = 0.340, *T*_max_ = 0.875
15574 measured reflections
2538 independent reflections
2436 reflections with *I* > 2σ(*I*)
*R*_int_ = 0.039

#### Refinement

*R*[*F* ^2^ > 2σ(*F* ^2^)] = 0.025
*wR*(*F* ^2^) = 0.061
*S* = 1.09
2538 reflections
204 parameters
H atoms treated by a mixture of independent and constrained refinement
Δρ_max_ = 0.69 e Å^−3^
Δρ_min_ = −0.45 e Å^−3^

### 

Data collection: *APEX2* (Bruker, 2007 ▶); cell refinement: *APEX2*; data reduction: *SAINT* (Bruker, 2007 ▶); program(s) used to solve structure: *SHELXS97* (Sheldrick, 2008 ▶); program(s) used to refine structure: *SHELXL97* (Sheldrick, 2008 ▶); molecular graphics: *SHELXTL* (Sheldrick, 2008 ▶); software used to prepare material for publication: *SHELXTL*.

## Supplementary Material

Crystal structure: contains datablocks I, global. DOI: 10.1107/S1600536808028195/pk2108sup1.cif

Structure factors: contains datablocks I. DOI: 10.1107/S1600536808028195/pk2108Isup2.hkl

Additional supplementary materials:  crystallographic information; 3D view; checkCIF report

## Figures and Tables

**Table 1:** Hydrogen-bond geometry (Å, °)

*D*—H⋯*A*	*D*—H	H⋯*A*	*D*⋯*A*	*D*—H⋯*A*
O1—H1*B*⋯O19^i^	0.73 (4)	2.01 (4)	2.727 (2)	169 (4)
O1—H1*A*⋯O20^ii^	0.75 (3)	1.95 (3)	2.695 (2)	169 (3)

Symmetry codes: (i) 
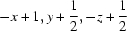
; (ii) 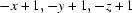.

**Fig. 1.**
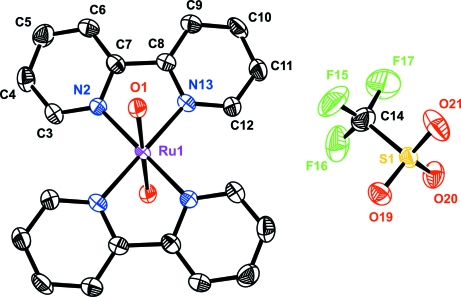
Single crystal structure of the title compound with 50% probability displacement ellipsoids. H atoms are omitted for clarity.

**Fig. 2.**
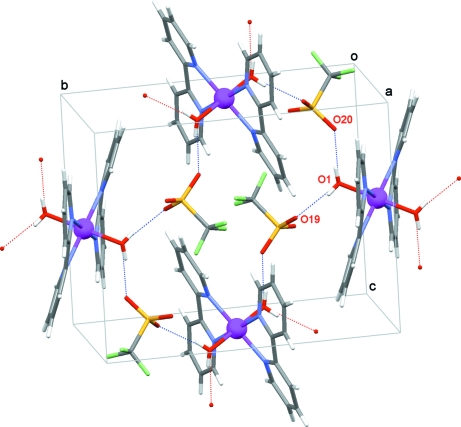
A packing diagram showing the hydrogen bonds (as dotted lines) between the water ligands in the ruthenium complex and the sulfonate group in the trifluoromethanesulfonate counterions.

## supplementary crystallographic information

### Comment

The water ligands in *cis*- and *trans*-[Ru(bpy)_2_(H_2_O)_2_]^2+^ can undergo facile ligand substitution under mild reaction conditions and thus these complexes have been important synthetic precursors to derivatives of the general type [Ru(bpy)_2_(*X*)(Y)]*^n^*. Although the *trans* isomer is usually obtained from the photo-induced isomerization of the more common *cis* isomer (Durham *et al.*, 1980; Walsh & Durham, 1982), formation of the *trans* isomer as a by-product in synthetic preparations involving the *cis*-Ru(bpy)_2_ moiety has also been reported (*e.g.* Weathers *et al.*, 1997).

The title compound *trans*-[Ru(bpy)_2_(H_2_O)_2_](CF_3_SO_3_)_2_, (I), was unintentionally obtained and crystallized from an acidic (0.1 *M* CF_3_SO_3_H) aqueous solution of [(bpy)_2_Ru(OMe)(pyz)Ru(bpy)_2_](PF_6_)_2_ (Jude *et al.*, 2008) that was left aging in ambient conditions for several weeks. Its crystal structure is shown in Figs. 1 and 2, and detailed structural information is available herein as *supplementary materials* as well as in the archived CIF.

The structure of the analogous compound as a hexafluorophosphate salt, *i.e. trans*-[Ru(bpy)_2_(H_2_O)_2_](PF_6_)_2_, (II), was previosuly reported (Weathers *et al.*, 1997). In this case, the compound crystallized in the triclinic (*P*1) space group. The Ru—O distance of 2.116 (2) Å in the PF_6_^-^ salt is similar to that reported here for the CF_3_SO_3_^-^ analogue, and the mean Ru—N distance of 2.074 (2) Å in II is essentially identical to those observed for I. The dihedral angle between the pyridyl (C_5_N) ring planes in the distorted, non-planar bpy ligands is also similar in these compounds: 162.68 (12)° for II and 160.1 (1)° for I.

Another related structure was reported earlier for the oxidized/deprotonated Ru(III)-hydroxy species, *trans*-[Ru(bpy)_2_(H_2_O)(OH)](ClO_4_)_2_ (Durham *et al.*, 1980). This perchlorate salt, (III), crystallized in the trigonal space group (*P*3_1_21). In this case, however, the sterically strained bpy ligands adopted a *twisted* conformation, in contrast to the *bowed* conformation in both I and II. Shorter Ru—N distances are exhibited by the Ru(II)-diaqua species compared to III (2.090 (3) and 2.099 (3) Å), owing to the characteristic π-backbonding involving Ru(II) and π-acceptor pyridyl ligands. Consistent with the higher Ru(III) oxidation state, however, the Ru—O bond distance is shorter in III (2.007 (3) Å).

The *trans*-[Ru(bpy)_2_(H_2_O)_2_]^2+^ cations pack in alternating layers with the CF_3_SO_3_^-^ anions packed between the cations. An extensive hydrogen bonding network exists within two-dimensional layers as a result of the hydrogen bonds between the water ligand molecules in the cationic complex and the sulfonate groups in the trifluoromethanesulfonate anions (Table 1 and Figure 2). However, no significant hydrogen-bond interactions are present between different layers.

### Experimental

*cis*-Ru(bpy)_2_Cl_2_ was prepared as described by Sullivan *et al.* (1978). The title compound was a product of the decomposition of [(bpy)_2_Ru(OMe)(pyz)Ru(bpy)_2_](PF_6_)_2_ (Jude *et al.*, 2008) in a highly acidic (0.1 *M* CF_3_SO_3_H) aqueous solution that was kept for several weeks in a sealed clear glass jar in ambient lighting and temperature conditions. Red–orange blocks suitable for single-crystal X-ray analysis were isolated from the mixture.

### Refinement

Diffraction data for single crystals of I were collected for 2θ < 139.4° on a Bruker AXS SMART APEXII diffractometer. The structure was solved by direct methods and hydrogen atoms were included in the final refinement cycles at predicted positions, with the exception of H1A and H1B which were refined isotropically.

### Crystal data

**Table:**

[Ru(C_10_H_8_N_2_)_2_(H_2_O)_2_](CF_3_O_3_S)_2_	*F*(000) = 748
*M**_r_* = 747.61	*D*_x_ = 1.796 Mg m^−^^3^
Monoclinic, *P*2_1_/*c*	Cu *K*α radiation, λ = 1.54178 Å
Hall symbol: -P 2ybc	Cell parameters from 1677 reflections
*a* = 8.6569 (5) Å	θ = 5.0–67.3°
*b* = 14.1272 (8) Å	µ = 6.88 mm^−^^1^
*c* = 11.3226 (6) Å	*T* = 100 K
β = 93.095 (3)°	Plate, brown
*V* = 1382.71 (13) Å^3^	0.20 × 0.15 × 0.02 mm
*Z* = 2	

### Data collection

**Table:**

Bruker SMART APEXII CCD area-detector diffractometer	2538 independent reflections
Radiation source: fine-focus sealed tube	2436 reflections with *I* > 2σ(*I*)
graphite	*R*_int_ = 0.039
φ and ω scans	θ_max_ = 69.7°, θ_min_ = 5.0°
Absorption correction: numerical Numerical followed by SADABS (Bruker, 2007)	*h* = −9→10
*T*_min_ = 0.340, *T*_max_ = 0.875	*k* = −17→17
15574 measured reflections	*l* = −13→13

### Refinement

**Table:**

Refinement on *F*^2^	Primary atom site location: structure-invariant direct methods
Least-squares matrix: full	Secondary atom site location: difference Fourier map
*R*[*F*^2^ > 2σ(*F*^2^)] = 0.025	Hydrogen site location: difference Fourier map
*wR*(*F*^2^) = 0.062	H atoms treated by a mixture of independent and constrained refinement
*S* = 1.09	*w* = 1/[σ^2^(*F*_o_^2^) + (0.0273*P*)^2^ + 1.4103*P*] where *P* = (*F*_o_^2^ + 2*F*_c_^2^)/3
2538 reflections	(Δ/σ)_max_ < 0.001
204 parameters	Δρ_max_ = 0.69 e Å^−^^3^
0 restraints	Δρ_min_ = −0.45 e Å^−^^3^

### Special details

**Table:**

Geometry. All e.s.d.'s (except the e.s.d. in the dihedral angle between two l.s. planes) are estimated using the full covariance matrix. The cell e.s.d.'s are taken into account individually in the estimation of e.s.d.'s in distances, angles and torsion angles; correlations between e.s.d.'s in cell parameters are only used when they are defined by crystal symmetry. An approximate (isotropic) treatment of cell e.s.d.'s is used for estimating e.s.d.'s involving l.s. planes.
Refinement. Refinement of *F*^2^ against ALL reflections. The weighted *R*-factor *wR* and goodness of fit *S* are based on *F*^2^, conventional *R*-factors *R* are based on *F*, with *F* set to zero for negative *F*^2^. The threshold expression of *F*^2^ > 2σ(*F*^2^) is used only for calculating *R*-factors(gt) *etc* and is not relevant to the choice of reflections for refinement. *R*-factors based on *F*^2^ are statistically about twice as large as those based on *F*, and *R*-factors based on ALL data will be even larger.Hydrogen atoms were placed in calculated positions with the exception of H1A and H1A, which were located in a difference synthesis and subsequently allowed to refine with isotropic thermal parameters.

### Fractional atomic coordinates and isotropic or equivalent isotropic displacement parameters (Å^2^)

**Table:**

	*x*	*y*	*z*	*U*_iso_*/*U*_eq_	
Ru1	0.5000	0.5000	0.0000	0.01303 (9)	
O1	0.64907 (19)	0.61137 (12)	0.05421 (17)	0.0181 (3)	
N2	0.6089 (2)	0.42131 (12)	0.13474 (15)	0.0154 (4)	
C3	0.5507 (3)	0.39407 (15)	0.23786 (19)	0.0192 (4)	
H3	0.4431	0.4020	0.2475	0.023*	
C4	0.6412 (3)	0.35501 (17)	0.3302 (2)	0.0243 (5)	
H4	0.5965	0.3365	0.4014	0.029*	
C5	0.7986 (3)	0.34355 (18)	0.3164 (2)	0.0271 (5)	
H5	0.8644	0.3208	0.3800	0.033*	
C6	0.8584 (3)	0.36563 (17)	0.2090 (2)	0.0230 (5)	
H6	0.9650	0.3559	0.1970	0.028*	
C7	0.7606 (3)	0.40225 (15)	0.11871 (19)	0.0179 (4)	
C8	0.8048 (2)	0.41339 (15)	−0.00489 (19)	0.0170 (4)	
C9	0.9491 (3)	0.38753 (16)	−0.0439 (2)	0.0208 (5)	
H9	1.0312	0.3708	0.0113	0.025*	
C10	0.9709 (3)	0.38668 (17)	−0.1642 (2)	0.0240 (5)	
H10	1.0692	0.3716	−0.1926	0.029*	
C11	0.8470 (3)	0.40816 (17)	−0.2426 (2)	0.0230 (5)	
H11	0.8578	0.4043	−0.3255	0.028*	
C12	0.7075 (3)	0.43527 (16)	−0.19861 (19)	0.0194 (4)	
H12	0.6233	0.4499	−0.2528	0.023*	
N13	0.6869 (2)	0.44164 (12)	−0.08110 (16)	0.0160 (4)	
S1	0.31188 (6)	0.31728 (4)	0.61491 (5)	0.01857 (13)	
C14	0.2290 (3)	0.3937 (2)	0.4986 (2)	0.0343 (6)	
F15	0.1904 (2)	0.34309 (16)	0.40134 (15)	0.0556 (5)	
F16	0.3298 (2)	0.45966 (12)	0.46821 (15)	0.0458 (4)	
F17	0.1035 (2)	0.43667 (17)	0.53333 (18)	0.0652 (6)	
O19	0.44116 (19)	0.27407 (11)	0.55926 (14)	0.0241 (4)	
O20	0.3551 (2)	0.38361 (12)	0.70808 (14)	0.0271 (4)	
O21	0.1893 (2)	0.25354 (14)	0.64049 (17)	0.0360 (5)	
H1A	0.644 (3)	0.6198 (19)	0.120 (3)	0.016 (7)*	
H1B	0.619 (4)	0.651 (2)	0.018 (3)	0.033 (9)*	

### Atomic displacement parameters (Å^2^)

**Table:**

	*U*^11^	*U*^22^	*U*^33^	*U*^12^	*U*^13^	*U*^23^
Ru1	0.01032 (13)	0.01596 (13)	0.01305 (13)	0.00058 (8)	0.00289 (8)	−0.00017 (7)
O1	0.0178 (8)	0.0206 (8)	0.0159 (9)	−0.0017 (6)	0.0017 (6)	0.0007 (7)
N2	0.0146 (9)	0.0163 (8)	0.0155 (9)	0.0011 (7)	0.0015 (7)	−0.0003 (7)
C3	0.0195 (11)	0.0191 (10)	0.0196 (11)	0.0000 (8)	0.0064 (9)	−0.0007 (8)
C4	0.0310 (13)	0.0241 (11)	0.0185 (11)	0.0024 (9)	0.0070 (10)	0.0042 (9)
C5	0.0291 (14)	0.0300 (12)	0.0219 (11)	0.0101 (10)	−0.0011 (10)	0.0028 (9)
C6	0.0164 (11)	0.0288 (12)	0.0240 (11)	0.0054 (9)	0.0022 (9)	0.0008 (9)
C7	0.0174 (11)	0.0169 (10)	0.0196 (11)	0.0008 (8)	0.0041 (9)	−0.0011 (8)
C8	0.0149 (11)	0.0164 (10)	0.0198 (11)	−0.0002 (8)	0.0016 (9)	−0.0010 (8)
C9	0.0163 (11)	0.0228 (11)	0.0232 (11)	0.0020 (9)	0.0018 (9)	−0.0001 (9)
C10	0.0180 (12)	0.0285 (12)	0.0265 (12)	0.0039 (9)	0.0102 (10)	0.0003 (9)
C11	0.0228 (12)	0.0281 (12)	0.0188 (11)	0.0033 (9)	0.0063 (9)	−0.0001 (9)
C12	0.0192 (12)	0.0227 (11)	0.0167 (10)	0.0016 (9)	0.0027 (9)	−0.0003 (8)
N13	0.0137 (9)	0.0160 (8)	0.0185 (9)	−0.0004 (7)	0.0037 (7)	−0.0005 (7)
S1	0.0180 (3)	0.0219 (3)	0.0161 (2)	−0.0016 (2)	0.0032 (2)	−0.00187 (19)
C14	0.0296 (15)	0.0488 (17)	0.0245 (13)	0.0170 (12)	−0.0003 (11)	0.0023 (11)
F15	0.0536 (12)	0.0841 (14)	0.0270 (9)	0.0107 (10)	−0.0172 (8)	−0.0078 (9)
F16	0.0665 (12)	0.0385 (9)	0.0332 (9)	0.0124 (8)	0.0110 (8)	0.0147 (7)
F17	0.0458 (11)	0.0919 (16)	0.0583 (12)	0.0474 (11)	0.0068 (9)	0.0086 (11)
O19	0.0218 (9)	0.0244 (8)	0.0266 (8)	0.0029 (6)	0.0054 (7)	0.0001 (6)
O20	0.0347 (10)	0.0275 (9)	0.0191 (8)	−0.0044 (7)	0.0015 (7)	−0.0040 (7)
O21	0.0340 (11)	0.0391 (10)	0.0366 (10)	−0.0155 (8)	0.0171 (8)	−0.0102 (8)

### Geometric parameters (Å, °)

**Table:**

Ru1—N2^i^	2.0727 (17)	C7—C8	1.479 (3)
Ru1—N2	2.0727 (17)	C8—N13	1.360 (3)
Ru1—N13^i^	2.0739 (17)	C8—C9	1.396 (3)
Ru1—N13	2.0739 (17)	C9—C10	1.385 (3)
Ru1—O1^i^	2.1053 (16)	C9—H9	0.9500
Ru1—O1	2.1053 (16)	C10—C11	1.389 (4)
O1—H1A	0.75 (3)	C10—H10	0.9500
O1—H1B	0.73 (4)	C11—C12	1.384 (3)
N2—C3	1.352 (3)	C11—H11	0.9500
N2—C7	1.363 (3)	C12—N13	1.355 (3)
C3—C4	1.387 (3)	C12—H12	0.9500
C3—H3	0.9500	S1—O21	1.4334 (18)
C4—C5	1.389 (4)	S1—O20	1.4451 (17)
C4—H4	0.9500	S1—O19	1.4486 (16)
C5—C6	1.383 (3)	S1—C14	1.820 (3)
C5—H5	0.9500	C14—F17	1.323 (3)
C6—C7	1.391 (3)	C14—F16	1.334 (3)
C6—H6	0.9500	C14—F15	1.340 (3)
			
N2^i^—Ru1—N2	180.00 (8)	N2—C7—C6	121.9 (2)
N2^i^—Ru1—N13^i^	77.18 (7)	N2—C7—C8	113.93 (19)
N2—Ru1—N13^i^	102.82 (7)	C6—C7—C8	123.8 (2)
N2^i^—Ru1—N13	102.82 (7)	N13—C8—C9	122.0 (2)
N2—Ru1—N13	77.18 (7)	N13—C8—C7	114.07 (18)
N13^i^—Ru1—N13	180.0	C9—C8—C7	123.6 (2)
N2^i^—Ru1—O1^i^	86.51 (7)	C10—C9—C8	119.0 (2)
N2—Ru1—O1^i^	93.49 (7)	C10—C9—H9	120.5
N13^i^—Ru1—O1^i^	86.88 (7)	C8—C9—H9	120.5
N13—Ru1—O1^i^	93.12 (7)	C9—C10—C11	119.0 (2)
N2^i^—Ru1—O1	93.49 (7)	C9—C10—H10	120.5
N2—Ru1—O1	86.51 (7)	C11—C10—H10	120.5
N13^i^—Ru1—O1	93.12 (7)	C12—C11—C10	119.3 (2)
N13—Ru1—O1	86.88 (7)	C12—C11—H11	120.4
O1^i^—Ru1—O1	180.0	C10—C11—H11	120.4
Ru1—O1—H1A	110 (2)	N13—C12—C11	122.4 (2)
Ru1—O1—H1B	102 (3)	N13—C12—H12	118.8
H1A—O1—H1B	113 (3)	C11—C12—H12	118.8
C3—N2—C7	117.74 (19)	C12—N13—C8	117.98 (18)
C3—N2—Ru1	127.77 (15)	C12—N13—Ru1	127.48 (15)
C7—N2—Ru1	114.28 (14)	C8—N13—Ru1	114.35 (14)
N2—C3—C4	122.9 (2)	O21—S1—O20	115.16 (10)
N2—C3—H3	118.6	O21—S1—O19	114.90 (11)
C4—C3—H3	118.6	O20—S1—O19	114.53 (10)
C3—C4—C5	118.6 (2)	O21—S1—C14	104.58 (13)
C3—C4—H4	120.7	O20—S1—C14	102.69 (12)
C5—C4—H4	120.7	O19—S1—C14	102.65 (11)
C6—C5—C4	119.2 (2)	F17—C14—F16	108.4 (2)
C6—C5—H5	120.4	F17—C14—F15	108.5 (2)
C4—C5—H5	120.4	F16—C14—F15	107.4 (2)
C5—C6—C7	119.2 (2)	F17—C14—S1	110.79 (19)
C5—C6—H6	120.4	F16—C14—S1	111.30 (19)
C7—C6—H6	120.4	F15—C14—S1	110.4 (2)
			
N13^i^—Ru1—N2—C3	16.01 (19)	C9—C10—C11—C12	3.8 (4)
N13—Ru1—N2—C3	−163.99 (19)	C10—C11—C12—N13	−0.1 (4)
O1^i^—Ru1—N2—C3	−71.58 (18)	C11—C12—N13—C8	−5.0 (3)
O1—Ru1—N2—C3	108.42 (18)	C11—C12—N13—Ru1	169.81 (17)
N13^i^—Ru1—N2—C7	−158.51 (14)	C9—C8—N13—C12	6.5 (3)
N13—Ru1—N2—C7	21.49 (14)	C7—C8—N13—C12	−166.89 (19)
O1^i^—Ru1—N2—C7	113.90 (15)	C9—C8—N13—Ru1	−169.01 (16)
O1—Ru1—N2—C7	−66.10 (15)	C7—C8—N13—Ru1	17.6 (2)
C7—N2—C3—C4	5.3 (3)	N2^i^—Ru1—N13—C12	−16.10 (19)
Ru1—N2—C3—C4	−169.07 (17)	N2—Ru1—N13—C12	163.90 (19)
N2—C3—C4—C5	0.2 (4)	O1^i^—Ru1—N13—C12	71.02 (18)
C3—C4—C5—C6	−4.1 (4)	O1—Ru1—N13—C12	−108.98 (18)
C4—C5—C6—C7	2.5 (4)	N2^i^—Ru1—N13—C8	158.85 (14)
C3—N2—C7—C6	−7.0 (3)	N2—Ru1—N13—C8	−21.15 (14)
Ru1—N2—C7—C6	168.14 (17)	O1^i^—Ru1—N13—C8	−114.03 (15)
C3—N2—C7—C8	166.29 (18)	O1—Ru1—N13—C8	65.97 (15)
Ru1—N2—C7—C8	−18.6 (2)	O21—S1—C14—F17	57.2 (2)
C5—C6—C7—N2	3.2 (3)	O20—S1—C14—F17	−63.4 (2)
C5—C6—C7—C8	−169.4 (2)	O19—S1—C14—F17	177.4 (2)
N2—C7—C8—N13	0.6 (3)	O21—S1—C14—F16	177.87 (17)
C6—C7—C8—N13	173.7 (2)	O20—S1—C14—F16	57.3 (2)
N2—C7—C8—C9	−172.6 (2)	O19—S1—C14—F16	−61.9 (2)
C6—C7—C8—C9	0.5 (3)	O21—S1—C14—F15	−63.0 (2)
N13—C8—C9—C10	−2.8 (3)	O20—S1—C14—F15	176.41 (18)
C7—C8—C9—C10	169.9 (2)	O19—S1—C14—F15	57.3 (2)
C8—C9—C10—C11	−2.4 (3)		

Symmetry codes: (i) −*x*+1, −*y*+1, −*z*.

### Hydrogen-bond geometry (Å, °)

**Table:**

*D*—H···*A*	*D*—H	H···*A*	*D*···*A*	*D*—H···*A*
O1—H1B···O19^ii^	0.73 (4)	2.01 (4)	2.727 (2)	169 (4)
O1—H1A···O20^iii^	0.75 (3)	1.95 (3)	2.695 (2)	169 (3)

Symmetry codes: (ii) −*x*+1, *y*+1/2, −*z*+1/2; (iii) −*x*+1, −*y*+1, −*z*+1.
